# The Real-Time Detection of Vertical Displacements by Low-Cost GNSS Receivers Using Precise Point Positioning

**DOI:** 10.3390/s24175599

**Published:** 2024-08-29

**Authors:** Aleksandra Maciejewska, Maciej Lackowski, Tomasz Hadas, Kamil Maciuk

**Affiliations:** 1 Institute of Geodesy and Geoinformatics, Wroclaw University of Environmental and Life Sciences, C.K. Norwida 25, 50-375 Wroclaw, Poland; 2 Department of Integrated Geodesy and Cartography, AGH University of Krakow, al. A. Mickiewicza 30, 30-059 Krakow, Poland

**Keywords:** GNSS levelling, real time, PPP, vertical displacements, low-cost GNSS

## Abstract

Vertical displacements are traditionally measured with precise levelling, which is inherently time consuming. Rapid or even real-time height determination can be achieved by the Global Navigation Satellite System (GNSS). Nevertheless, the accuracy of real-time GNSS positioning is limited, and the deployment of a network of continuously operating GNSS receivers is not cost effective unless low-cost GNSS receivers are considered. In this study, we examined the use of geodetic-grade and low-cost GNSS receivers for static and real-time GNSS levelling, respectively. The results of static GNSS levelling were processed in four different software programs or services. The largest differences for ellipsoidal/normal heights reached 0.054 m/0.055 m, 0.046 m/0.047 m, and 0.058 m/0.058 m for points WRO1, BM_ROOF, and BM_CP, respectively. In addition, the values depended on the software used and the location of the point. However, the multistage experiment was designed to analyze various strategies for GNSS data processing and to define a method for detecting vertical displacement in a time series of receiver coordinates. The developed method combined time differentiation of coordinates estimated for a single GNSS receiver using the Precise Point Positioning (PPP) technique and Butterworth filtering. It demonstrated the capability of real-time detection of six out of eight displacements in the range between 20 and 55 mm at the three-sigma level. The study showed the potential of low-cost GNSS receivers for real-time displacement detection, thereby suggesting their applicability to structural health monitoring, positioning, or early warning systems.

## 1. Introduction

These days, the Global Navigation Satellite System (GNSS) has become an indispensable component of modern life, largely without consideration of its underlying structure or its diverse range of applications. It is a ubiquitous technology, employed in a multitude of contexts, including navigation [1], tourism [2,3], aviation [4], life-saving [5], and precision agriculture [6]. High-accuracy GNSS is predominantly utilized in geodetic applications, such as coordinate determination [7] and plate tectonics [8], surveying and seismology [9], and atmosphere monitoring [10]. Recently, significant attention has been directed toward the development of low-cost GNSS receivers. Although a limited accuracy was achieved with the first single-frequency low-cost GNSS receivers, which were not able to eliminate ionosphere delay [11], dual-frequency low-cost GNSS receivers have been successfully applied in, e.g., land surveying, during favorable survey conditions [12], and normal height determination [13]. However, it is widely recognized that geodetic-grade GNSS receivers and antennas offer superior performance compared to low-cost hardware [14].

In the vast majority of low-cost GNSS demonstrations, two positioning techniques are used: Precise Point Positioning (PPP) and real-time kinematics (RTK). For a long time, the PPP technique was associated with post-processing, thus rendering it unsuitable for (near) real-time applications. Its principal benefit was sub-centimeter accuracy and mm-level precision, achievable by a single GNSS receiver. On the other hand, RTK is a real-time technique but requires a (network of) nearby reference station and its accuracy is limited to 2–3 cm horizontally and 5 cm vertically [15]. Therefore, RTK is not suitable for detecting small displacements, particularly in the vertical direction. Furthermore, the use of RTK in any open areas like underdeveloped countries, seas, or airspace is not feasible due to the lack of an appropriate infrastructure of the system. Concerning Standard Point Positioning (SPP), this method does not achieve the required accuracy [16] and only PPP can come up to the expectations and is the most suitable solution. The mathematical model of the PPP technique is less complex than RTK. However, PPP necessitates precise products and modeling of satellite, receiver, and site displacement effects. Moreover, the highly correlated receiver height, receiver clock error, and troposphere delays result in a long initialization time [17,18]. The PPP accuracy is additionally susceptible to the observation interval, selected orbit and clock products, and the weighting function [19]. Despite these limitations, PPP remains a prospective technique that is still being improved and its applications are still broadening. It is applied in atmosphere monitoring [20], UAV photogrammetry [21], precision agriculture [22], engineering structures [23], and land monitoring [24]. To overcome PPP limitations, the International GNSS Service (IGS) launched the Real-Time Service (RTS) in 2013. The RTS provides satellite orbits and clock corrections, thus enabling real-time PPP. However, the limited accuracy when compared with the post-processing mode is noteworthy. Initially, a sub-decimeter accuracy was achieved [25], but with the subsequent development of real-time products [26], real-time static PPP has achieved accuracy comparable to RTK [27].

The occurrence of natural hazards, weather phenomena, and environmental effects makes it necessary to monitor the condition of civil structures such as buildings, dams, tunnels, and bridges [28]. Furthermore, a point displacement refers to a change in point position in regard to the established reference frame over a determined time interval [29]. However, a deformation is defined as the sum of the displacement and strain of an object. Furthermore, it represents a change in mutual positions of the points without a dilated object lump that does not affect material continuity. To ensure the monitoring of constant changes in the aforementioned objects, control surveys are conducted. The frequency of these surveys is dependent upon the requirements of the construction project manager, the nature of the object in question, and the prevailing legislation. Classic geodetic techniques, such as total stations and geometric levelling, are still applicable today [30]. The authors combined these methods to achieve the best results and accuracy, ensuring that the root mean square (RMS) uncertainty did not exceed 3.0 mm over a distance of 100 m. Furthermore, GNSS can be utilized as the sole source of information to monitor displacements. In addition, the displacements can be measured in real time with the application of the Trimble correction service [31]. The research was focused on assessing dynamic changes for early warning or rapid risk assessment, yielding an average RMS error (RMSE) of 3.6 mm. Moreover, the PPP technique was adopted in conjunction with five constellations (GPS, Galileo, GLONASS, BDS, QZSS) for structural health monitoring [32]. The study proved that integrating diverse systems can enhance the accuracy and reduce the sheltering effect.

The relationship between PPP and vertical displacement has already been established, e.g., for wave height monitoring during hurricanes [33]. The authors analyzed results from kinematic PPP and PPK (post-processing kinematic) and proved the accuracy of kinematic PPP with a 10 cm RMS error. Dynamic vertical displacement detection using PPP was consistent with relative positioning at a 10 mm level and the high-rate kinematic PPP method can capture the vertical natural frequency of engineering structures [34]. A static PPP method allows for vertical structural motion monitoring even at a 1–2 mm level [35]. The PPP method has also been used as a tool for vertical crustal deformation detection [36], determining the ocean tide vertical displacement parameter [37], or in various PPP scenarios for determining the vertical displacement [38].

Low-cost antennas and receivers are available in a variety of sizes and shapes, which underscores their potential to be used in a multitude of applications. Additionally, the antennas are compatible with multiple signal bands, including various satellite systems, which makes them a cost-effective and versatile alternative to geodetic-grade antennas. These factors render low-cost GNSS devices a viable alternative to geodetic-grade GNSS receivers, thereby reducing the financial burden associated with conducting observations and procuring necessary equipment [39]. Moreover, it was demonstrated that the RTK solution can provide accurate results when implemented in low-cost equipment on the order of 5.5 mm and 11 mm for horizontal and vertical components, respectively [40]. Furthermore, a comparative analysis of the results obtained from the CSRS-PPP service in both static and kinematic modes was conducted [41]. The evaluation also included the geodetic receiver, which provided coordinates and served as a reference. The use of PPP static mode with a high sampling rate resulted in centimeter-level precision, making it an appropriate choice for structural health monitoring and urban positioning. What is more, the low-cost GNSS receivers were used to build a network in order to monitor integrated water vapor (IWV) [42]. The computations were performed both during post-processing and in real time using PPP static mode. The standard deviations of IWV differences in comparison to the water vapor radiometer were found to be 1.0 and 1.5 kg/m^2^ in post-processing and real time, respectively. Researchers are developing low-cost GNSS equipment with the objective of replacing geodetic-grade hardware in continuous surveys and everyday usage. Consequently, their architecture, processing methods, and applications are still being expanded and enhanced.

This study aims to develop a method that allows the detection of vertical displacements in real time with low-cost GNSS receivers, with a focus on magnitudes below the typical vertical precision of the RTK technique, i.e., 5 cm (1 sigma). Moreover, since the GNSS levelling procedures are not standardized, we compare height determination results from selected software and techniques. The paper is organized as follows. Initially, we introduce the PPP technique, low-cost equipment, Butterworth filtering, processing of GNSS data, and the conducted field work. Subsequently, we compare GNSS levelling results and demonstrate the robustness of the proposed methods for displacement detection. Finally, a summary and conclusions of the obtained information are provided, along with indications of potential future developments.

## 2. Materials and Methods

### 2.1. Precise Point Positioning

PPP is a positioning technique that primarily distinguishes itself by using both the code and carrier phases from a single GNSS receiver [43]. The utilization of the carrier phase enables the attainment of superior precision compared to the majority of alternative techniques, including RTK. However, the primary distinction between PPP and these techniques is that PPP is absolute, necessitating the usage of a single receiver without any reference stations and requiring simultaneous observations [44]. The precision of the position determination is subordinated to precise products such as precise satellite orbits and clocks [45]. All of these factors can provide solutions at the centimeter level. Furthermore, it can be improved to the millimeter level in post-processing or static mode [46].

The original model of the PPP technique was presented in 1997 to overcome the computational complexity of global networks caused by the radical growth in GNSS observations [43]. As an alternative, it is sufficient to determine the precise orbits and clocks of only a specified group of globally deployed GNSS stations. The calculated orbits and clocks can be used to independently determine the position of other stations. The known parameters are fixed in the observing system, while receiver position and clock error can be determined using pseudorange (code) and carrier-phase observations at two frequencies [47]:

(1) $$P_{IF}^{s}=\frac{1}{\;f_{1}^{2}-f_{2}^{2}}\left(f_{1}^{2}P_{1}^{s}-f_{2}^{2}P_{2}^{s}\right)=\rho_{0}+e_{r}^{s}\cdot \delta X_{r}+c\left(\delta t_{r}-\delta t^{s}\right)+\delta T_{r}^{s}+\epsilon_{P}^{s}+\Delta P_{IF}^{s}$$

(2) $$L_{IF}^{s}=\left[\frac{1}{\;f_{1}^{2}-f_{2}^{2}}\left(\;f_{1}^{2}L_{1}^{s}-f_{2}^{2}L_{2}^{s}\right)\right]\lambda_{IF}=\rho_{0}+e_{r}^{s}\cdot \delta X_{r}+c\left(\delta t_{r}-\delta t^{s}\right)+\delta T_{r}^{s}+\lambda_{IF}\cdot N_{IF}^{s}+\epsilon_{L}^{s}+\Delta L_{IF}^{s}$$

where:

$P_{IF}$—ionosphere-free linear combination of code-phase observations [m];
$L_{IF}$—ionosphere-free linear combination of carrier-phase observations [m];
s—number of satellites;
$P_{1/2}^{s},\;L_{1/2}^{s}$—code- and carrier-phase observations for both frequencies [m];
$f_{1},f_{2}$—carrier frequency [Hz];
$\rho_{0}$—geometrical range from the receiver to the antenna [m];
c—the vacuum speed of light [m/s];
$\delta t^{s},$ $\delta t_{r}$—satellite and receiver clock errors [s];
$\delta T_{r}^{s}$—the signal path delay due to the neutral atmosphere [m];
$N_{IF}^{s}$—phase ambiguity parameter in ionosphere-free combination [cyc.];
$\lambda_{IF}$—carrier-phase wavelength in ionosphere-free combination [m];
$\epsilon_{P}^{s},\;\epsilon_{L}^{s}$—noise measurement in code- and carrier-phase observations [m];
$e_{r}^{s}$—vector of angular coefficients [.];
$\delta X_{r}$—vector of corrections for the receiver’s position [m];
$\Delta P_{IF}^{s}$/$\Delta L_{IF}^{s}$—code- and carrier-phase corrections for the receiver, satellite, and tidal effects in ionosphere-free combination [m].

Alternatively, undifferenced and uncombined observations can be processed under the ionospheric delay-constrained (IC-PPP) model [48,49]:

(3) $$P_{i}^{s}=\rho_{0}+e_{r}^{s}\cdot \delta X_{r}+c\left(\delta t_{r}+\delta t^{s}\right)+\delta T_{r}^{s}+\frac{f_{1}^{2}}{f_{i}^{2}}\cdot I^{s}+\Delta P_{i}^{s}$$

(4) $$L_{i}^{s}=\rho_{0}+e_{r}^{s}\cdot \delta X_{r}+c\left(\delta t_{r}+\delta t^{s}\right)+\delta T_{r}^{s}-\frac{f_{1}^{2}}{f_{i}^{2}}\cdot I^{s}+\lambda_{i}\cdot N_{i}^{s}+\Delta L_{i}^{s}$$

where:

$\lambda_{i}$—original length of signal for *i* frequency [m];
$N_{i}^{s}$—integer phase ambiguity parameter;
$\Delta P_{i}^{s},\;\Delta L_{i}^{s}$—code- and carrier-phase corrections for the receiver, satellite, and tidal effects for *i*-th frequency [m];
$I^{s}$—slant ionospheric delay [m].

In contrast to the original PPP model, the ionospheric delays must be estimated in IC-PPP, which is possible because the system of equations is built up to twice the size of observations than in the ionosphere-free model. The main advantage of the IC-PPP model is the possibility of regaining complete (integer) information about the phase ambiguity [50], which leads to a fixed solution, fast initialization, and high accuracy.

### 2.2. Low-Cost GNSS Hardware

**Figure 1:**
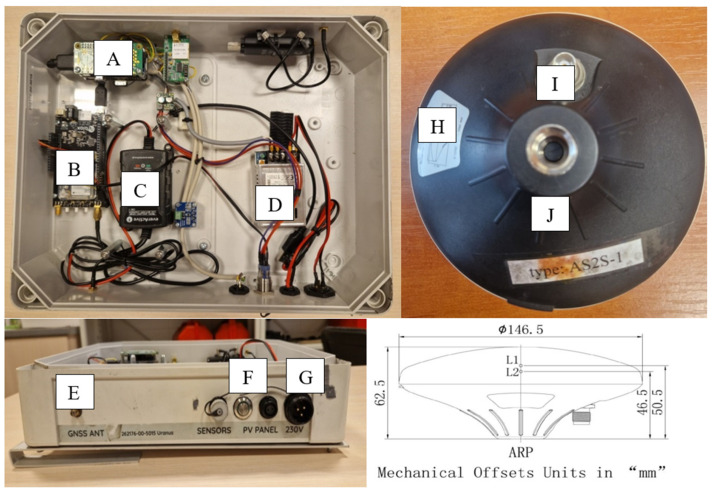
Components of the low-cost GNSS receiver and antenna: A—microcomputer, B—GNSS module, C—power supply, D—LED power supply, E—GNSS antenna socket, F—LED switch, G—power socket, H—sticker (https://www.ardusimple.com/product/survey-gnss-multiband-antenna/, access: 11 May 2024), I—TNC socket, J—UNC socket.

The prototype low-cost GNSS receiver was equipped with a microcomputer Raspberry Pi Zero W V1.1 and high-precision GNSS module u-blox ZED-F9P (Figure 1). The chipset tracks the L1 and L2 signals of GPS and E1 and E5b signals from Galileo. The receiver was connected to ArduSimple 2-band antennas, for which the antenna phase center offsets for L1 and L2 are provided. No calibration for the E5b signal was available, thus the L2 offset was applied. The GNSS observations were recorded with a 5 s interval in the RINEX 3.04 format.

### 2.3. Butterworth Filter

Data filtering is a component that is designed to reduce the amount of information transmitted by eliminating data that are either redundant or erroneous [51]. However, the development of technology constituted a significant breakthrough, enabling researchers to use computers for real-time data filtering. The Butterworth filter is a digital filter based on the time domain [52]. It is characterized by maximally flattening the magnitude of the signal. The main goal of Butterworth’s idea was to develop an analogue low-pass filter that could exemplify the ideal signal. The generalized equation representing the Butterworth filter is as follows [53]:

(5) $$H_{\left(j\omega \right)}=\frac{1}{\sqrt{1+\varepsilon^{2}{\left(\frac{\omega }{\omega_{c}}\right)}^{2n}}}$$

where:

$H_{\left(j\omega \right)}$—the frequency response;
*n*—order of the filter;
ω—operating frequency of the circuit;
$\omega_{c}$—cut-off frequency;
*ε*—maximum passband gain.

The Butterworth filter is commonly used to filter GNSS and inertial signals [54,55], and high-rate GNSS products [56,57], hence its applications for GNSS-based detection of deformation is investigated in this study. The second-order Butterworth filter is considered in this study due to its capability to maximally flatten the signal within the passband in comparison to other filters.

### 2.4. GNSS Data Processing

The GNSS data were processed using the GNSS-WARP software [27]. The algorithm was implemented in the MATLAB (MATrix LABoratory, Dubai, United Arab Emirates) environment from scratch at Wroclaw University of Environmental and Life Sciences (UPWr, Wrocław, Poland) by a research team from the Institute of Geodesy and Geoinformatics. The software has been adapted to process static and kinematic multi-GNSS data in both real-time and post-processing modes. In real-time mode, it requires communication with the BKG Ntrip Client (BNC) software v2.x to decode real-time observations and corrections transmitted through the Networked Transport of Radio Technical Commission for Maritime (RTCM) via Internet Protocol (NTRIP).

We employed the PPP technique and the simulated real-time mode, using real-time multi-GNSS orbits and clocks from the Centre National D’Etudes Spatiales (CNES). Kalman filtering was used to estimate the vector state, including receiver coordinates. For the BX15 station, static coordinates were estimated, whereas BX08 coordinates were considered kinematic. In the kinematic case, both tight (0.01 m) and loose (1 m) constraining of coordinates were considered. Moreover, since the vertical component is sensitive to the cut-off angle, two settings were investigated, i.e., 10° and 20°.

### 2.5. Field Work

**Figure 2:**
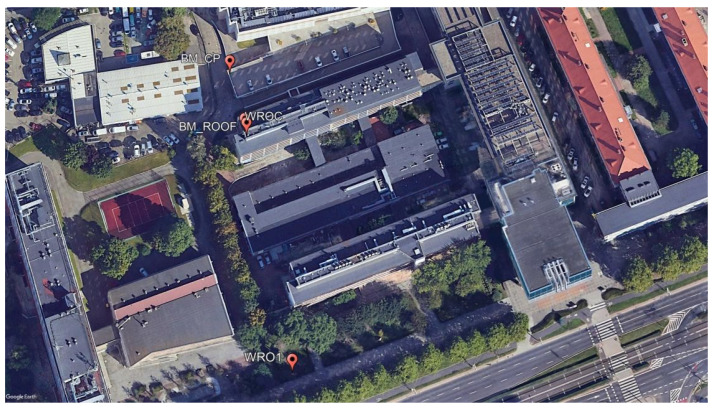
The test base set by precise levelling (source: Google Earth).

We designed the test base and established it by precise levelling. The test base was located around and on the roof of the C1 Building of the UPWr (Figure 2). The main aim of the precise levelling was to obtain the normal heights (H, Table 1) of established benchmarks with the greatest possible degree of accuracy, thereby facilitating subsequent surveys. The survey was conducted with an accuracy of 1.3 mm, i.e., we obtained such a difference between the leveling and known height of the WROC station.

The next step was to measure the points WRO1, BM_CP, and BM_ROOF by geodetic-grade GNSS receivers in static mode with the objective being to achieve the highest possible accuracy and calculate their ellipsoidal heights (h). The GNSS survey was conducted for twelve hours, with an interval of 30 s. The following satellite systems were included: GPS, Galileo and GLONASS. Receiver coordinates were determined (1) in post-processing mode using the CSRS-PPP online service (https://webapp.csrs-scrs.nrcan-rncan.gc.ca/geod/tools-outils/ppp.php, access: 15 November 2023), (2) with static PPP processing in the GNSS-WARP software, (3) with static baseline mode in the Leica Geo Office (LGO) software v8.4, and (4) using the POZGEO online service of the ASG-EUPOS (https://system.asgeupos.pl/, access: 15 November 2023) system. Ultimately, ellipsoidal heights were measured twice by the real-time networking (RTN) technique and the arithmetic mean was calculated. For each method, we calculated the geoid undulation (N) with the corresponding (or built into the software) geoid model and checked the uncertainty of the height component.

**Figure 3:**
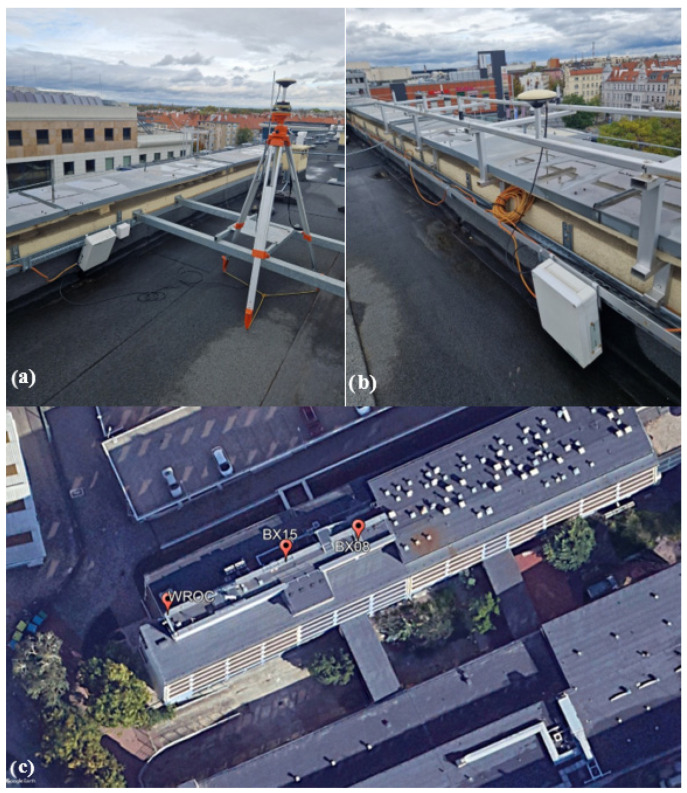
Experiment setup: (**a**) BX08 moving receiver, (**b**) BX15 static receiver, (**c**) location of receivers.

For the second part of the experiment, we used two low-cost GNSS receivers and antennas, named BX08 and BX15. Both receivers were positioned on the roof (open sky conditions) at a distance of several meters from each other (Figure 3). The interval of observations was 5 s and the following signals were tracked: GPS (L1 C/A, L2C), Galileo (E1 C, E5 b), and GLONASS (L1OF, L2OF). The BX15 receiver remained static, whereas the BX08 was used to manually simulate vertical displacements with an interval of 15 min. The amplitudes of these displacements ranged from 2 mm to 55 mm and were made in two different directions (up and down) in order to obtain the initial height at the end of the simulation of displacement. The survey was conducted on 30 October 2023 and the displacement simulation lasted approximately five hours (https://data.mendeley.com/datasets/w3hr2dgt2s/1 (accessed on 31 July 2024)).

### 2.6. Time-Series Variants

**Figure 4:**
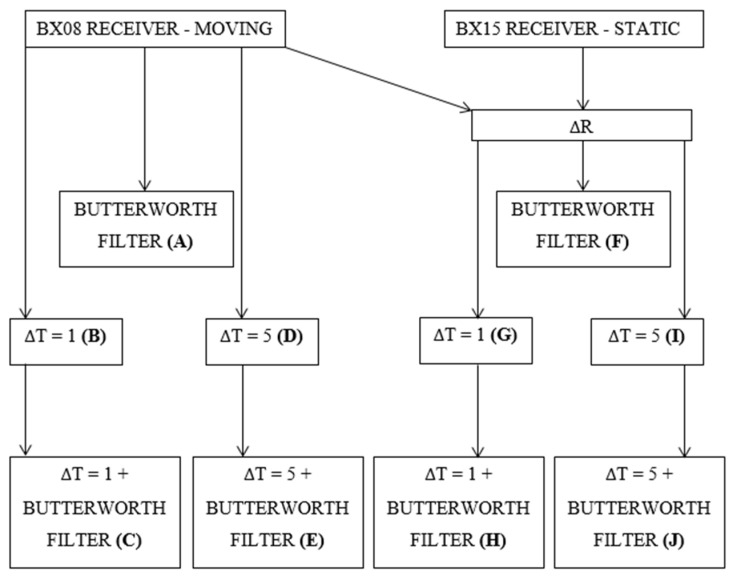
Definition and relations between time-series variants.

The vertical displacement detection was performed by the analysis of the time series of the vertical component or its derived products. Overall, ten different products (labeled A to J) were subjected to analysis. These time series were formed by the combination of time differentiation (ΔT, by 1 or 5 epochs), receiver differentiation (ΔR, between BX08 and BX15), and Butterworth filtering. The Butterworth filter high-pass setting was 0.01 Hz and was adjusted arbitrarily based on the previous experience of the authors. The rationale and labeling are explained in Figure 4.

## 3. Results

This research involved multistage measurement and analysis. Due to this fact, this section is divided into two parts that are separately focused on the measurements concerning GNSS levelling and the survey of vertical displacements, but also their analyses.

### 3.1. GNSS Levelling

The ellipsoidal/normal heights varied between software and methods (Table 2 and Table 3), with the largest differences reaching 0.054 m/0.055 m, 0.046 m/0.047 m, and 0.058 m/0.058 m for points WRO1 (RTN and POZGEO), BM_ROOF (CSRS-PPP and LGO), and BM_CP (CSRS-PPP and POZGEO), respectively. However, in most cases, the differences were below the uncertainty level. Formal errors at 1σ varied from 0.003 m (BM_CP from LGO) to 0.018 m (WRO1 from CSRS-PPP). Formal errors varied not only between software (e.g., from 0.003 m to 0.014 m for BM_CP in LGO and CSRS-PPP, respectively) but also between points processed by the same software (e.g., from 0.003 m to 0.013 m in LGO). Differences were also found between the geoid models implemented behind each software, but they remained below the 1 mm level. Such results indicate that GNSS leveling is highly sensitive to the GNSS data processing strategy. Therefore, it should be carefully selected for further applications in EWS.

### 3.2. Displacement Detection

#### 3.2.1. Impact of Selected Processing Parameters on Coordinate Time Series

**Figure 5:**
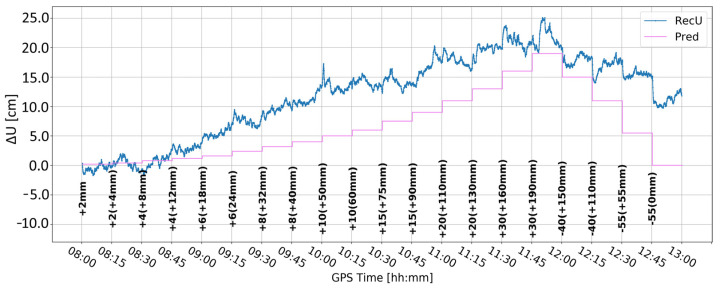
Height component estimated with elevation cut-off angle of 10° and 0.01 m coordinate constraints; BX08 receiver.

**Figure 6:**
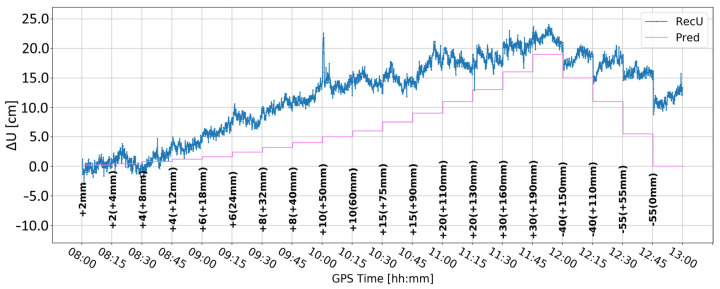
Height component estimated with elevation cut-off angle of 10° and 1 m coordinate constraints; BX08 receiver.

Figure 5 and Figure 6 present the change in the height component (∆U) with the application of two different levels of coordinate constraints, i.e., 0.01 (tight) and 1 m (loose), respectively. The loose constraints make the time series noisier and less similar to predicted displacements (Pred), drawn as the pink line, presenting the expected change in the height component. On the other hand, tight constraints resulted in a smoother time series, which changed rapidly during the simulated displacements of tens of mm. Moreover, the PPP filter excluded several GPS satellites around 10:00 and 12:00, resulting in large peak/discontinuity in the time series, which is suppressed in the tightly constrained solution. Therefore, it was concluded to continue with tight constraining of the coordinates.

**Figure 7:**
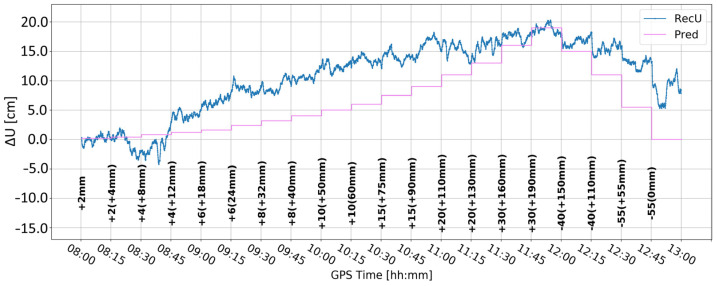
Height component estimated with elevation cut-off angle of 20° and 0.01 m coordinate constraints; BX08 receiver.

Figure 7 presents the change in the vertical component when the elevation cut-off angle is set to 20°. Compared to the 10° elevation cut-off angle (Figure 5), the time series is closer to the expected position. Nevertheless, at 8:30 an unexpected fault appears, which is bigger than in Figure 5, even though the values in the range 11:45–12:00 are the same for both lines. Moreover, the character of the time series obtained with a 10° elevation cut-off angle seems to better reflect the expected deformations. Therefore, it was arbitrarily decided to proceed with a 10° elevation cut-off angle.

#### 3.2.2. Time-Series Analysis

Ten time series (A–J) of the vertical component were delivered (Figure 4). For each time series the standard deviation was calculated and the resulting one-sigma, two-sigma, and three-sigma ranges were determined, which correspond to confidence levels of 68.3%, 95.4%, and 99.7%, respectively. It was assumed that a displacement at a specific confidence level occurred if the time series exceeded the corresponding sigma range. Therefore, the deformation detection relied on determining peaks (points) that were outside of the specific range. We also investigated so-called false alarms, which are positive responses of the detection algorithm (in this case, exceedance of a certain sigma level), that occur for non-displacement epochs.

**Figure 8:**
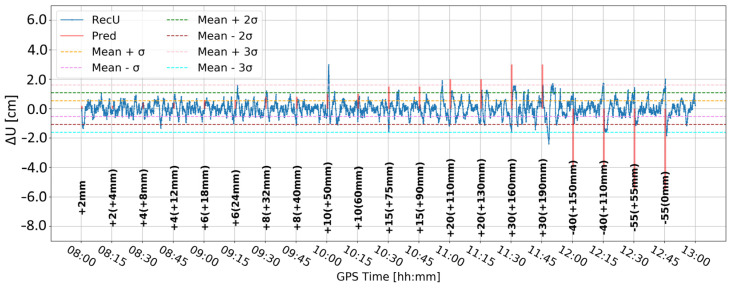
Time series A—signal after Butterworth filter.

**Figure 9:**
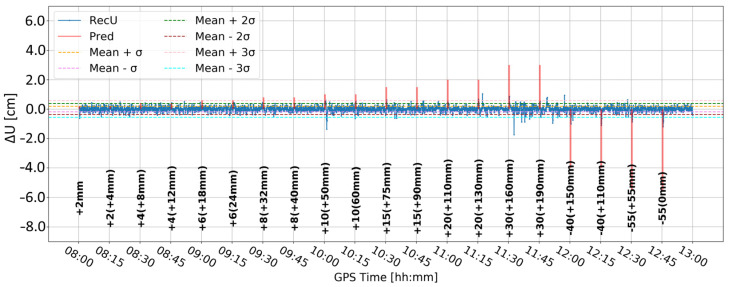
Time series B—signal after differencing by one epoch.

**Figure 10:**
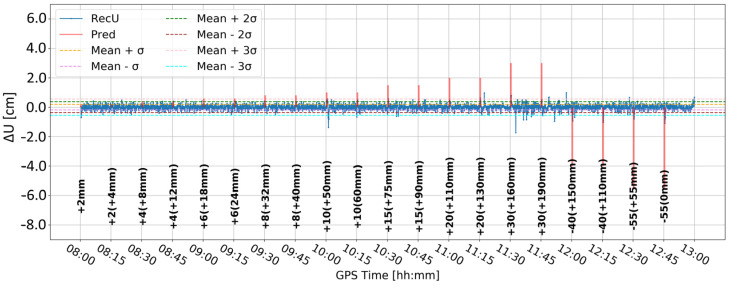
Time series C—signal after differencing by one epoch and Butterworth filter.

Time series A (Figure 8) was obtained by Butterworth filtering of the original time series. It was the one in which large variation in the vertical component is clearly visible. Some large deformation seemed to be visually detectable. However, since time series A was noisy, it was difficult to clearly distinguish between correct and false alarms. Time series B (Figure 9) resulted from time differentiation by one epoch (5 s). Several peaks appeared that exceeded the three-sigma level, but only some of them coincided with the real deformation epochs. The combined product, i.e., time series C (Figure 10), merged both methods, i.e., firstly the time series was differenced, and then, filtered, remained similar to time series B, suggesting that time differentiation by one epoch impacted the time series more than filtering.

**Figure 11:**
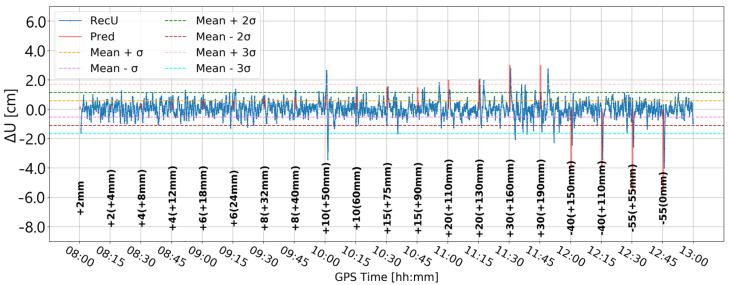
Time series D—signal after differencing by five epochs.

**Figure 12:**
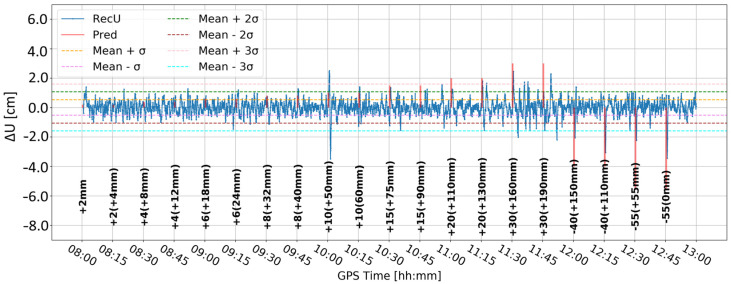
Time series E—signal after differencing by five epochs and Butterworth filter.

Figure 11 and Figure 12 present analogous solutions to the ones presented in Figure 9 and Figure 10. The only difference involved changing the time-differentiation interval from one epoch to five epochs (25 s). Such a change was motivated by the Kalman filter characteristics and stochastic modeling, which may prevent immediate update of the coordinates. As expected, the resulting time series had a wider range of values and larger sigma levels. Both time series are similar to each other. Nevertheless, multiple peaks coincided with the real deformation epochs, particularly for deformation with amplitudes larger than 10 mm. Moreover, the number of false alarms was reduced in both cases. Such results suggested that a larger differentiation time helps to detect deformation, while the impact of Butterworth filtering is marginal.

Another idea for detecting the displacements was making use of a nearby GNSS receiver, i.e., BX15, that was not affected by simulated displacements. While the idea is similar to RTK, there are fundamental differences between the two methods. While RTK assumes multiple error sources to cancel out by double differencing on the observation level, our approach operates on coordinates obtained with rigorous error modeling in the PPP technique, which are subject to further subtraction (moving minus static receiver). It was expected that such subtraction would reduce the impact of satellite geometry and the limited accuracy of real-time orbit and clock products. Nevertheless, due to the different locations of receivers, the multipath effect was amplified [58].

**Figure 13:**
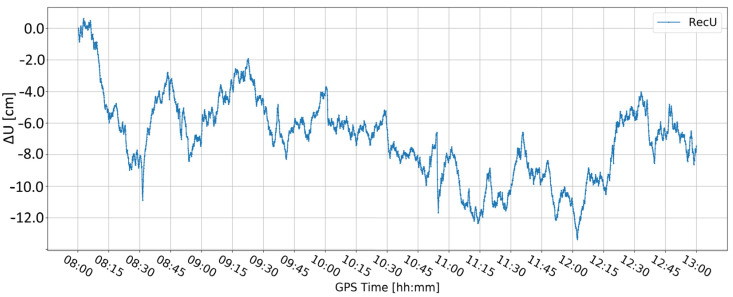
Height component estimated with elevation cut-off angle of 10° and 0.01 m coordinate constraining; BX15 receiver.

**Figure 14:**
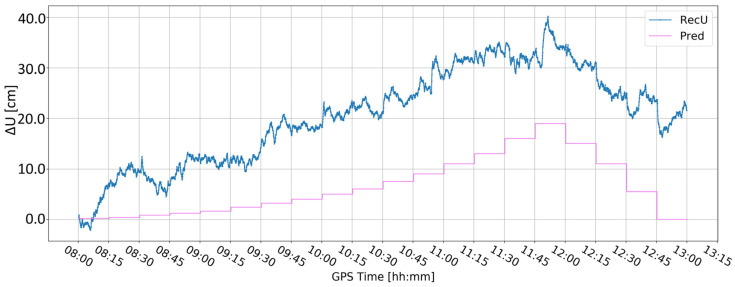
Height component estimated with elevation cut-off angle of 10° and 0.01 m coordinate constraining; difference between BX08 and BX15 (∆R).

**Figure 15:**
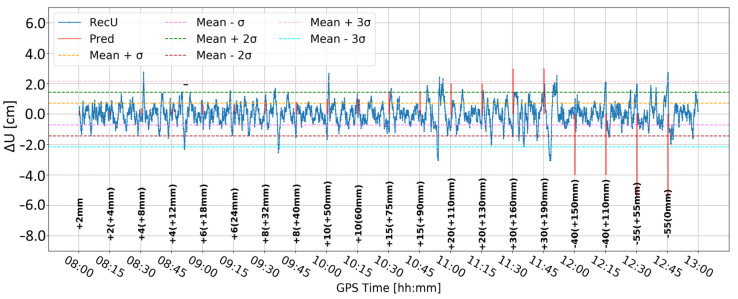
Time series F—∆R after Butterworth filter.

**Figure 16:**
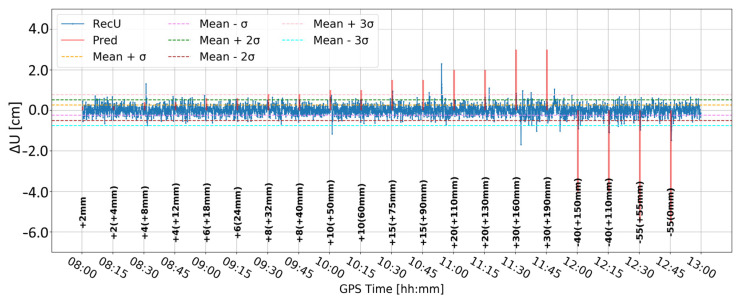
Time series G—∆R after differencing by one epoch.

**Figure 17:**
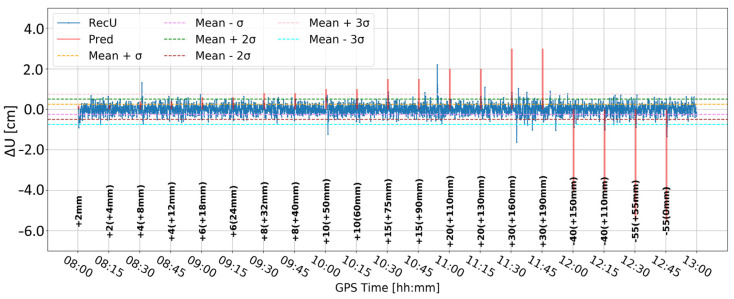
Time series H—∆R after differencing by one epoch and Butterworth filter.

**Figure 18:**
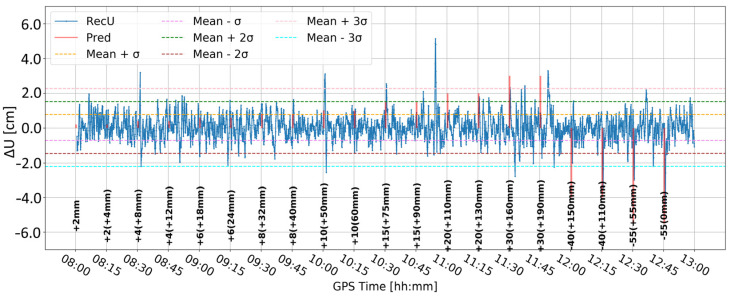
Time series I—∆R after differencing by five epochs.

**Figure 19:**
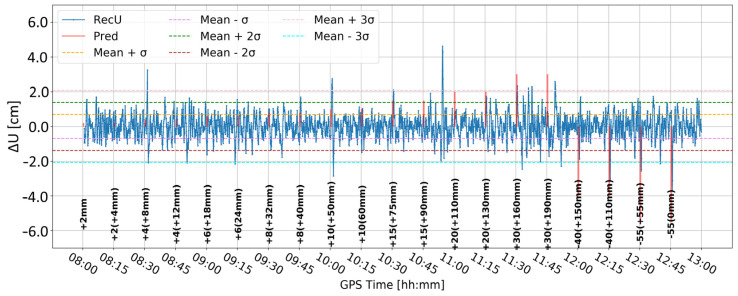
Time series J—∆R after differencing by five epochs and Butterworth filter.

Figure 13 presents the original time series of the vertical component for the static BX15 receiver, whereas Figure 14 presents the time series of differentiated heights. Even though the BX15 receiver was static, its real-time estimated height (by means of the Kalman filter) changed significantly. Therefore, differencing between receivers (variants F to J) impacted the resulting time series and was the subject of justified consideration. The corresponding time series are presented in Figure 15, Figure 16, Figure 17, Figure 18 and Figure 19.

Surprisingly, by differencing between two receivers, degradation rather than improvement was obtained. The major discontinuities (or, in other words, rapid changes) in the vertical component for the BX15 receiver were the reason for multiple additional peaks causing additional false alarms (e.g., around 8:31, 10:01, and 10:48). The previously undetected deformations remained invisible.

#### 3.2.3. Classification of Events

To clearly indicate the best-performing variant, the visual assessment is followed by a quantitative approach. The following events are considered:

1. Detected (DET.)—when displacement was simulated and a corresponding peak was detected by the algorithm;
2. False alarm (F.A.)—when a peak was detected by the algorithm but there was no simulated displacement at this time;
3. Undetected (UNDET.)—when the displacement was simulated, but it was not detected by the algorithm; this can be easily calculated by subtracting DET. from the total number of simulated displacements (20).

It is assumed, that the peak corresponds to the simulated displacement if its detection epoch varies by up to 30 s from the epoch of simulated displacement, as noted in the field. Such time-shifted response is justified by the limited accuracy and precision of the watch used in the field, the time needed to shift the receiver, the lag of the tightly constrained Kalman filter response, and the character of the algorithm (time differentiation). The time-scales (leap seconds in particular) were unified prior to the experiment.

Finally, the following ratios were proposed to reflect the robustness of the proposed methods:

(6) $$ratio_{det}=\frac{\;detected}{detected+FA}$$

(7) $$ratio_{undet}=\frac{undetected}{20\;}$$

(8) $$ratio_{FA}=\frac{FA}{detected+FA}$$

Table 4 contains the count of events and corresponding ratios for all variants (A–J) and the one-, two-, and three-sigma levels. The lower the confidence level was, not only were more displacements correctly detected but also more false alarms appeared, reaching over 190 for time series B, C, and H for the one-sigma level. For the three-sigma level, a maximum of 15 false alarms appeared for time series C. On the other hand, for time series D and E, there were 7 detected displacements (ratio of 0.54), 13 undetected displacements, and only 6 false alarms. For time series I the numbers were six (0.50) detected and six false alarms. The difference between D and E appeared at the two-sigma level, for which the time series E resulted in significantly fewer false alarms (34 instead of 44). Therefore, this method was considered the best among all ten variants.

For time series E and the three-sigma level, the following displacements were detected: 10 mm (10:00), 20 mm (11:15), 30 mm (11:30), 40 mm (12:00 and 12:15), and 55 mm (12:30 and 12:45). Therefore, for displacements of 20 mm or more, only two, 20 mm (11:00) and 30 mm (11:45), displacements remain undetected. However, the second one is followed by two false alarms (approximately 4 and 7 min later).

**Figure 20:**
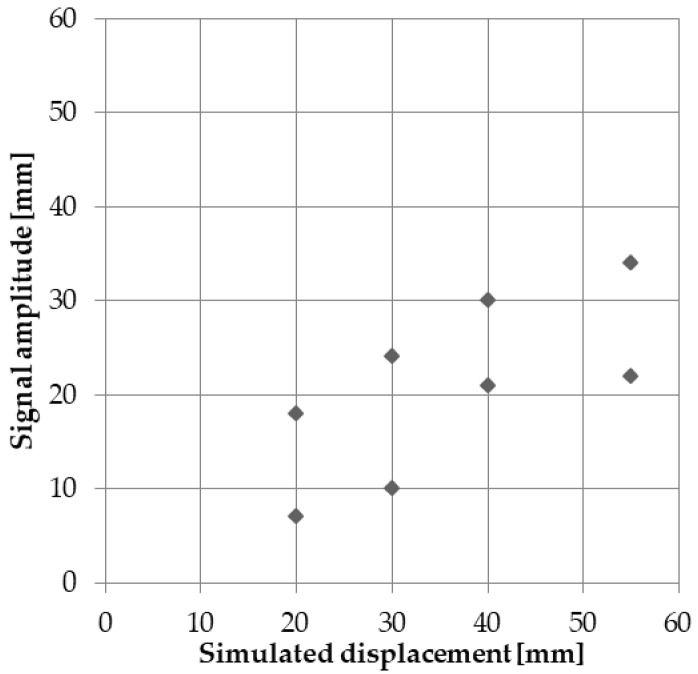
Relation between simulated values and those detected by the algorithm.

Last but not least, it is noted that the peak amplitudes of the detected displacements do not reflect the displacement value (Figure 20). Therefore, the proposed method is incapable of determining the amplitude of the displacement.

## 4. Summary

Real-time PPP and low-cost GNSS receivers are considered promising tools for structural health monitoring or early warning systems. This study aimed to (1) identify the accuracy of real-time satellite leveling with low-cost GNSS receivers, and (2) develop a method for detecting vertical displacement in a time series of receiver coordinates. The prototype low-cost GNSS receivers were used together with original real-time PPP software (among others) to prepare field experiments, including the simulation of vertical displacement ranging from 2 mm to 55 mm.

The GNSS leveling proved to be highly sensitive to the GNSS data processing strategy, i.e., different software estimated varying heights. The differences reached up to 58 mm, but in most cases remained below the uncertainty level (1σ) of a dozen mm. Therefore, it is not recommended to detect low-amplitude vertical displacement using real-time GNSS leveling. Height determination results obtained with different software should be compared with special care, as this leads to further inaccuracies in the estimates.

To detect vertical displacement in real-time GNSS PPP, ten strategies for time-series analysis were proposed. The strategies combined time differentiation (by one or five epochs), differentiation between receivers, and Butterworth filtering. We investigated the impact of coordinate constraining in the Kalman filter and the cut-off angle. The best results were obtained when the time series obtained from a tightly constrained (0.01 m) solution with an elevation cut-off angle of 10° was differentiated over time by five epochs, followed by the high-pass Butterworth filtering. The overall effectiveness at the three-sigma level (99.9%) showed the detection of the following displacements: seven properly classified, thirteen undetected, and six false alarms. However, only one displacement greater than 10 mm remained undetected, indicating that the proposed variant is robust for detecting displacements of 20 mm or more (seven out of eight events were detected), while still suffering from false alarms and being unable to detect the amplitude of displacements.

The findings of this research provide valuable insights for the development of an early warning system for small-scale displacements, which are often challenging to monitor using traditional techniques or prove not to be cost effective. It was demonstrated, that GNSS observations from low-cost receivers can be processed using a real-time PPP technique with accuracy comparable, if not superior, to RTK, as the proposed method was capable of detecting vertical displacements that were below the nominal vertical accuracy of the RTK technique. However, with regard to computed differences (3.1) and inventing an algorithm that detects displacements but not their amplitudes (3.2), there is no possibility to determine the specific accuracy of real-time satellite levelling with low-cost GNSS receivers.

Future studies should further investigate the impact of processing parameters on real-time determination of the receiver height and detection of statistically significant discontinuities in time series. Considering that the displacements are not expected to occur multiple times per day in real-case scenarios, a field experiment should be repeated with displacements simulated with a longer time interval to allow for a moving-window analysis.

## Figures and Tables

**Table 1:** Normal heights (PL-EVRF2007-NH) of points WRO1 and WROC.

Point Name	H (m)
WRO1	117.1331
WROC (measured)	140.7275
WROC (catalog)	140.7288

**Table 2:** Results from various software (h—ellipsoidal height, H—normal height, 1σ—uncertainty, N—geoid undulation).

Source	Point	h [m]	H [m]	1σ [mm]	N [m]
LGO	WRO1	157.2369	117.1460	±13	40.0909
	BM_ROOF	180.1167	140.0281	±6	40.0886
	BM_CP	162.7319	122.6432	±3	40.0887
POZGEO	WRO1	157.2250	117.1338	±11	40.0912
	BM_ROOF	180.1240	140.0358	±12	40.0882
	BM_CP	162.7150	122.6268	±12	40.0882
CSRS-PPP	WRO1	157.2710	117.1804	±18	40.0906
	BM_ROOF	180.1630	140.0747	±9	40.0883
	BM_CP	162.7730	122.6846	±14	40.0884
GNSS-WARP	WRO1	157.2612	117.1706	±6	40.0906
	BM_ROOF	180.1486	140.0603	±5	40.0883
	BM_CP	162.7443	122.6559	±7	40.0884

**Table 3:** Results from RTN (control) survey.

Point	Height Type	1st Survey [m]	2nd Survey [m]	Mean [m]	N [m]
WRO1 WRO1	Normal	117.1840	117.1930	117.1890	40.0909
	Ellipsoidal	157.2830	157.2750	157.2790	
BM_ROOF BM_ROOF	Normal	140.0310	140.0470	140.0390	40.0891
	Ellipsoidal	180.1200	180.1360	180.1280	
BM_CP BM_CP	Normal	122.6480	122.6610	122.6550	40.0886
	Ellipsoidal	162.7368	162.7494	162.7430	

**Table 4:** Number and ratio for three confidence levels (1σ, 2σ, and 3σ) and ten variants (A–J). Ratios are color coded from dark red (small), through yellow (medium), to dark green (large).

		1σ			2σ			3σ		
		DET.	UNDET.	F.A.	DET.	UNDET.	F.A.	DET.	UNDET.	F.A.
A	Number	6	14	121	9	11	29	3	17	5
	Ratio	0.05	0.70	0.95	0.24	0.55	0.76	0.38	0.85	0.63
B	Number	12	8	191	16	4	73	10	10	14
	Ratio	0.06	0.40	0.94	0.18	0.20	0.82	0.42	0.50	0.58
C	Number	11	9	194	16	4	70	10	10	15
	Ratio	0.05	0.45	0.95	0.19	0.20	0.81	0.40	0.50	0.60
D	Number	16	4	164	12	8	44	7	13	6
	Ratio	0.09	0.20	0.91	0.21	0.40	0.79	0.54	0.65	0.46
E	Number	14	6	166	11	9	34	7	13	6
	Ratio	0.08	0.30	0.92	0.24	0.45	0.76	0.54	0.65	0.46
F	Number	7	13	123	5	15	30	1	19	9
	Ratio	0.05	0.65	0.95	0.14	0.75	0.86	0.10	0.95	0.90
G	Number	13	7	186	15	5	79	6	14	12
	Ratio	0.07	0.35	0.93	0.16	0.25	0.84	0.33	0.70	0.67
H	Number	13	7	193	16	4	79	7	13	13
	Ratio	0.06	0.35	0.94	0.17	0.20	0.83	0.35	0.65	0.65
I	Number	11	9	163	11	9	38	6	14	6
	Ratio	0.06	0.45	0.94	0.22	0.45	0.78	0.50	0.70	0.50
J	Number	9	11	163	12	8	42	6	14	10
	Ratio	0.05	0.55	0.95	0.22	0.40	0.78	0.38	0.70	0.63

## Data Availability

The original contributions presented in the study are included in the article/Supplementary Material, further inquiries can be directed to the corresponding author/s.

## Supplementary Materials

The following supporting information can be downloaded at: https://www.mdpi.com/1424-8220/24/17/5599/s1, Reference materials are available in public repository at https://data.mendeley.com/datasets/w3hr2dgt2s/1 (accessed on 31 July 2024).
